# Adjusted morbidity groups and survival: a retrospective cohort study of primary care patients with chronic conditions

**DOI:** 10.1186/s12875-023-02059-9

**Published:** 2023-04-20

**Authors:** Mariana Bandeira-de Oliveira, Teresa Aparicio-González, Isabel del Cura-González, Carmen Suárez-Fernández, Ricardo Rodríguez-Barrientos, Jaime Barrio-Cortes

**Affiliations:** 1 Ciudad Jardin Health Centre, Primary Care Management, Madrid, Spain; 2 Goya Health Centre, Primary Care Management, Madrid, Spain; 3 Research Unit. Primary Care Management, Madrid, Spain; 4 Gregorio Marañón Health Research Institute, Madrid, Spain; 5 Department of Medical Specialties and Public Health, Rey Juan Carlos University, Madrid, Spain; 6 Research Network on Chronicity, Primary Care and Prevention and Health Promotion, Carlos III Health Institute, Madrid, Spain; 7 University Hospital of La Princesa, Madrid, Spain; 8 Department of Medicine, Autonomous University of Madrid, Madrid, Spain; 9 Foundation for Biosanitary Research and Innovation in Primary Care, Madrid, Spain; 10 Faculty of Health, Camilo José Cela University, Madrid, Spain

**Keywords:** Chronic conditions, Primary care, Multimorbidity, Risk levels, Survival

## Abstract

**Background:**

Chronic conditions are one of the main determinants of frailty, functional disability, loss of quality of life and the number one cause of death worldwide. This study aimed to describe the survival of patients with chronic conditions who were followed up in primary care according to the level of risk by adjusted morbidity groups and to analyse the effects of sex, age, clinician and care factors on survival.

**Methods:**

This was a longitudinal observational study of a retrospective cohort of patients with chronic conditions identified by the adjusted morbidity group stratifier of the electronic medical records in a primary health centre of the Region of Madrid, which has an assigned population of 18,107 inhabitants. The follow-up period was from June 2015 to June 2018. A description of survival according to the Kaplan–Meier method and Cox proportional hazards multivariate regression model was used to analyse the effects of sex, age, clinician and care factors.

**Results:**

A total of 9,866 patients with chronic conditions were identified; 77.4% (7,638) had a low risk, 18.1% (1,784) had a medium risk, and 4.5% (444) had a high risk according to the adjusted morbidity groups. A total of 477 patients with chronic conditions died (4.8%). The median survival was 36 months. The factors associated with lower survival were age over 65 years (hazard ratio [HR] = 1.3; 95% confidence interval [CI] = 1.1–1.6), receiving palliative care (HR = 3.4; 95% CI = 2.6–4.5), high versus low risk level (HR = 2.4; 95% CI = 1.60–3.7), five chronic conditions or more (HR = 1.5; 95% CI = 1.2-2), complexity index (HR = 1.01; 95% CI = 1.02–1.04) and polymedication (HR = 2.6; 95% CI = 2.0-3.3).

**Conclusions:**

There was a gradual and significant decrease in the survival of patients with chronic conditions according to their level of risk as defined by adjusted morbidity groups. Other factors, such as older age, receiving palliative care, high number of chronic conditions, complexity, and polymedication, had a negative effect on survival. The adjusted morbidity groups are useful in explaining survival outcomes and may be valuable for clinical practice, resource planning and public health research.

**Supplementary Information:**

The online version contains supplementary material available at 10.1186/s12875-023-02059-9.

## Introduction

The prevalence of chronic conditions is increasing as the populations of Western countries age [1, 2]. In Spain, the percentage of people over 65 years of age is 19.2% of the total population, which will rise to 25.2% in 2033 [3]. Patients with chronic conditions have higher mortality rates than patients without chronic conditions [4–8]. Chronic conditions and ageing are two of the main determinants of frailty, functional disability and loss of quality of life, leading to a series of pathophysiological, social, and health care-related events that increase the risk of death [4, 5].

Population stratification tools such as the Clinical Risk Group (CRG) or Adjusted Clinical Groups (ACG) are used to stratify the population and optimize clinical-care management, favouring a better distribution of resources and more efficient and patient-centred care [9, 10]. In recent years, adjusted morbidity groups (AMGs) have been implemented by the Spanish Health Ministry recommendation in primary care electronic clinical records in most of the Autonomous Communities of the country with the aim of improving and personalizing the management of patients with chronic conditions according to their risk levels [11, 12]. These risk levels are calculated through the assignment of cut-off points from a complexity index calculated by the stratification tool for the entire population. The influence of this grouping system on clinicians is large, and it is very often used because the tool is automatically integrated and extended in the electronic medical record, highlighting in the system each patient risk level to all primary care professionals [13]. In the Region of Madrid, these AMG risk levels compel them to choose different levels of interventions and therefore the care plan that each patient needs. Patients at a low-risk level are patients with mild chronic conditions or still in incipient stages. For this goal, self-management and health education are promoted to avoid the progression of the health condition and health care utilization. Medium-risk patients are patients who suffer chronic conditions that need a more disease-based approach. The objective at this level is to slow progression by combining self-management and professional care of the health condition. High-risk patients are complex patients with multimorbidity who need a multidisciplinary care approach and extensive health care services. The goal at this level is to increase survival and to reduce exacerbations, complications, emergency room visits and hospital admissions through comprehensive case management, with mainly professional care [14].

Although there is growing population-based evidence about the impact of AMG morbidity and complexity on survival in Catalonia, there is still limited evidence on the impact of the complexity index and the risk level by AMG on survival in specific cohorts of patients with chronic conditions in primary-care follow-up in other regions of Spain [12, 15–17], as stratification in different autonomous communities could not be comparable [13]. This work provides useful and novel information to better explain the AMG complexity index and risk levels in a clinical practice setting. The objective of this study was to describe the survival of a cohort of patients with chronic conditions followed up in primary care in the Region of Madrid according to the level of risk by AMG and to assess the independent effects of sex, age, and other clinician and care factors on survival.

## Methods

This was a retrospective observational study of a follow-up cohort. The study ran from June 30, 2015, to June 29, 2018. The study area was a primary health centre of the Chamartín district of Madrid, with an assigned population of 18,107 people. In this area, the population is served by a health centre made up of professionals from primary care doctors, paediatricians, nurses, social workers, dentists, physiotherapists, and administrative support staff. The study subjects consisted of people identified as having one or more chronic conditions by the AMG classification tool included in the Madrid primary care electronic medical record (AP-Madrid). This tool identified all patients of any age who presented with at least one of the chronic conditions described in Additional File 1 as of June 30, 2015 (ICPC-2 codes that were considered relevant chronic conditions according to the Care Strategy for people with chronic conditions promoted by the Department of Health of the Region of Madrid) [14].

The input for the AMG algorithm is a text file comprising data about the diagnosis (health problems) of the patients. Each record in the input file corresponds to a health condition. The required domains are identification of the patient, diagnostic classification used, code of the diagnosis, date of diagnosis, sex and birthdate of the patient. Instead of considering all possible diagnostic codes individually, the AMG tool creates a diagnostic code group (DCG), which gathers all codes associated with a given chronic condition. AMG classifies the population into 31 mutually exclusive categories based on both morbidity and complexity. Complexity is calculated from three relevant information blocks corresponding to individuals with the given DCG: (1) morbidity, (2) health care needs (i.e., visits to primary care and hospital admissions), and (3) prescriptions and is converted for each patient into a numerical value or “complexity index” [12]. These weight values have been obtained by modelling DCG and outcome data from the catchment population of the Catalan Health care System (data collected in 2011 from 7.5 million people). Since the complexity index is a continuous variable, putting this index into percentiles allows the stratification of each individual of the population, allocating them into four risk groups following the model of the Kaiser-Permanente pyramid [18] (high-risk patients with chronic conditions above the 95th percentile, medium-risk patients with chronic conditions between the 85th and 95th percentiles, patients with chronic conditions in the low risk level between the 50th and 85th percentiles and patients without relevant chronic conditions below the 50th percentile). These AMGs have been elaborated and subsequently analysed with data from the general population [12, 13, 15–17, 19] and specifically checked on different types of populations [20–22].

The information was extracted from the AP-Madrid program as of June 30, 2015, and age and sex were collected as sociodemographic variables. As clinician and care variables, the following were collected: immobilization institutionalization, primary caregiver, home support, receiving palliative care risk level by AMG, complexity index by AMG [12], multimorbidity, type of chronic conditions presented and polymedication. The information on mortality from any cause recorded in the clinical history was retrieved during a 3-year period (June 30, 2015, to June 29, 2018). Variables studied are enumerated and detailed in Table 1.

**Table 1:** Variables

Age	Quantitative *years*
Sex	Categorical *male/female*
Immobilization	Qualitative *yes/no*, considered as spending most of the time in bed or having considerable difficulty in moving around (which prevents them from leaving their home, except in exceptional cases) for any reason and who foreseeable duration of this difficulty is greater than two months
Institutionalization	Qualitative *yes/no*, considered as staying in nursing home/retirement home
Primary caregiver	Qualitative *yes/no*, considered as having a caregiver at home
Home support	Qualitative *yes/no*
Receiving palliative care	Qualitative *yes/no*
Risk level by AMG	Qualitative *low/medium/high*
Complexity index by AMG	Quantitative numerical value of patient complexity assigned by AMG which is an index measured as a function of morbidity and health service utilization
Multimorbidity	Qualitative *yes/no* considered as the simultaneous presence of 2 or more chronic conditions in a patient that require a comprehensive and multidisciplinary approach [23–25]
Chronic conditions (presented in Additional File 1).	Qualitative *yes/no*
Polymedication	Qualitative *yes/no*, considered as patients with medication regimen that implies having been prescribed five or more medications for their chronic conditions as a reference treatment
Death	Qualitative *yes/no*

AMG: Adjusted morbidity groups

Survival was evaluated from the start date, which corresponded to the date of risk stratification (the same for all patients) until the end of follow-up, the patient’s death, or the end of the study.

There were no missing or incomplete data at the moment of the data extraction because variables were fully registered in AP-Madrid. However, a total of 150 patients were lost to follow-up because they moved to another region and they were censored in the analysis. In these cases, the follow-up ended on the date of the last contact recorded in the clinical history.

A univariate descriptive analysis was performed with frequencies and percentages for the qualitative variables and with means and standard deviations for the quantitative variables. In the bivariate analysis, the chi-squared test was applied to compare qualitative variables. The nonparametric Mann–Whitney U test was used for between-group comparisons. Median survival and survival curves were produced using Kaplan–Meier analysis. The factors associated with survival were studied with Cox multivariate regression analysis, with the dependent variable being the survival time in months. We explored by bivariate analysis which factors were associated with survival, and the independent variables with a level of significance lower than 0.05 were included in the Cox multivariate regression. A threshold of 5 or more concurrent chronic conditions was established instead of the cut-off point from two (qualitative) or the number of chronic conditions (quantitative) in the analysis because five is a threshold previously used in the literature to define more complex patients (9–15). The 95% confidence intervals were calculated for hazard ratios. The Wald test was used to assess differences between variables and survival in the Cox analysis. Confounding was evaluated in the multivariate analysis by the stratification method, and interactions between explanatory variables were explored. To analyse the data and draw graphs, SPSS version 25 was used.

The study was approved by the Local Research Commission of the Centre Teaching Unit and the Ethics Committee of Drug Research of La Princesa University Hospital.

## Results

A total of 9,866 patients with chronic conditions were identified, corresponding to 54.4% of the population assigned to the health centre. Table 2 shows the sociodemographic and clinical-care characteristics of chronic patients and their differences segmented by sex and level of risk. The mean age of the patients with chronic conditions was 55.7 years, 61.4% were women, and 3% were immobilized. Their mean number of chronic conditions was 2.5, with 61.2% multimorbidity and 16.2% polymedication. Regarding sex, females presented higher average age, higher prevalence of immobilization, higher multimorbidity and higher polymedication. These differences between sexes were statistically significant (p ≤ 0.05). According to the stratification by AMG, 77.4% of patients with chronic conditions had a low risk level, 18.1% had medium risk, and 4.5% had high risk. There were more females in the low-risk group than in the medium-risk and high-risk groups. The median age was lower in the low-risk group than in the medium- and high-risk groups. Low risk presented less average chronic conditions than medium and high risk. The complexity index mean was significantly inferior in the low-risk group than in the medium- and high-risk groups. Polymedication was observed less frequently among patients with low risk than among those with medium and high risk. These differences between risk levels were statistically significant (p ≤ 0.01).

**Table 2:** Sociodemographic, clinician and care variables of patients with chronic conditions and differences by sex and level of risk

Variables n (%)	Total 9,866 (100)	Females 6,056 (61.4)	Males 3,810 (38.6)	p value^a^	Low risk 7,638 (77.4)	Medium risk 1,784 (18.1)	High risk 444 (4.5)	p value^b^
Female sex	6,056 (61.4)	-	-	-	4,665 (61.1)	1,159 (65.0)	232 (52.3)	< 0.01
Age*	55.7 (20.8)	57.1 (20.7)	53.5 (20.7)	< 0.01	50.6 (19.4)	72.1 (15.1)	77.8 (13.0)	< 0.01
Immobilized	300 (3.0)	223 (3.7)	77 (2.0)	< 0.01	49 (0.6)	126 (7.1)	125 (28.2)	< 0.01
Institutionalized	161 (1.6)	122 (2.0)	39 (1.0)	< 0.01	67 (0.9)	52 (2.9)	42 (9.5)	< 0.01
Primary caregiver	229 (2.3)	164 (2.7)	65 (1.7)	< 0.01	26 (0.3)	101 (5.7)	102 (23.0)	< 0.01
Home support	80 (0.8)	58 (1.0)	22 (0.6)	0.05	13 (0.2)	38 (2.1)	29 (6.5)	< 0.01
Palliative care	44 (0.4)	20 (0.3)	24 (0.6)	0.04	8 (0.1)	7 (0.4)	29 (6.5)	< 0.01
Complexity index *	6.7 (7.0)	6.7 (6.5)	6.7 (7.8)	< 0.01	4 (2.2)	12.4 (2.7)	30.4 (12.5)	< 0.01
Chronic conditions *	2.5 (1.8)	2.62 (1.9)	2.3 (1.8)	< 0.01	1.9 (1.1)	4.3 (1.6)	6.7 (2.4)	< 0.01
1 chronic condition 2 chronic conditions 3 chronic conditions 4 chronic conditions 5 chronic conditions > 5 chronic conditions	3,830 (38.8) 2,185 (22.1) 1,487 (15.1) 1,021 (10.3) 609 (6.2) 734 (7.5)	2,225 (36.7) 1,319 (21.8) 951 (15.7) 663 (10.9) 413 (6.8) 485 (8.0)	1,605 (42.1) 866 (22.7) 536 (14.1) 358 (9.4) 196 (5.1) 249 (6.4)	< 0.01 0.269 0.027 0.014 0.001 0.007	3,763 (49.3) 2,034 (26.6) 1,096 (14.3) 537 (7.0) 170 (2.2) 38 (0.5)	63 (3.5) 141 (7.9) 365 (20.5) 445 (24.9) 383 (21.5) 387 (21.7)	4 (0.9) 10 (2.3) 26 (5.9) 39 (8.8) 56 (12.6) 309 (69.6)	< 0.01 < 0.01 < 0.01 < 0.01 < 0.01 < 0.01
Multimorbidity	6,036 (61.2)	3,831 (63.3)	2,205 (57.9)	< 0.01	3,875 (50.7)	1,721 (96.5)	440 (99.1)	< 0.01
Polymedicated	1,598 (16.2)	1,101 (18.2)	497 (13.0)	< 0.01	473 (6.2)	774 (43.4)	351 (79.1)	< 0.01

*$\overline X$ (SD). ^a^ p value shows the differences between males and females based on bivariate analysis. ^b^ p value shows the differences between risk levels based on bivariate analysis

Table 3 shows the main chronic conditions and differences segmented by sex and level of risk. The most prevalent chronic conditions were arterial hypertension (34.6%), obesity (16.5%), depression (12.7%) and diabetes mellitus (10.8%). Some conditions were more prevalent among females, such as depression (15.8% versus 7.7%) and dementia (2.7% versus 1.3%), whereas other conditions were more prevalent among men: arterial hypertension (37.3% versus 33.0%), diabetes mellitus (13.6% versus 9.0%), cirrhosis (6.2% versus 4.0%), ischaemic heart disease (6.1% versus 2.3%), neoplasias (6.1% versus 4.1%) and chronic obstructive pulmonary disease (5.8% versus 2.8%). These differences between sexes were statistically significant (p ≤ 0.01). At the low-risk level, the most prevalent conditions were hypertension (24.3%), obesity (13.5%) and depression (10.0%). The medium-risk levels were arterial hypertension (67.2%), obesity (26%) and diabetes (24.2%). At the high-risk level, the most prevalent conditions were hypertension (82.0%), diabetes mellitus (42.6%) and neoplasia (37.6%). These differences between risk levels were statistically significant (p < 0.01).

**Table 3:** Main chronic conditions and differences by sex and level of risk

Variables n (%)	Total 9,866 (100)	Female 6,056 (61.4)	Male 3,810 (38.6)	p- value^a^	Low risk 7,638 (77.4)	Medium risk 1,784 (18.1)		High risk 444 (4.5)	p value^b^
Arterial hypertension	3,418 (34.6)	1,998 (33.0)	1,420 (37.3)	< 0.01	1,855 (24.3)		1,199 (67.2)	364 (82.0)	< 0.01
Chronic heart failure	240 (2.4)	151 (2.5)	89 (2.3)	0.64	16 (0.2)		101 (5.7)	123 (27.7)	< 0.01
Chronic renal insufficiency	142 (1.4)	70 (1.2)	72 (1.9)	< 0.01	9 (0.1)		36 (2.0)	97 (21.8)	< 0.01
Cirrhosis	479 (4.9)	241 (4.0)	238 (6.2)	< 0.01	241 (3.2)		188 (10.5)	50 (11.3)	< 0.01
COPD	389 (3.9)	168 (2.8)	221 (5.8)	< 0.01	115 (1.5)		165 (9.2)	109 (24.5)	< 0.01
Dementia	213 (2.2)	162 (2.7)	51 (1.3)	< 0.01	64 (0.8)		93 (5.2)	56 (12.6)	< 0.01
Depression	1,251 (12.7)	957 (15.8)	294 (7.7)	< 0.01	764 (10.0)		386 (21.6)	101 (22.7)	< 0.01
Diabetes mellitus	1,063 (10.8)	546 (9.0)	517 (13.6)	< 0.01	442 (5.8)		432 (24.2)	189 (42.6)	< 0.01
Ischaemic heart disease	370 (3.8)	137 (2.3)	233 (6.1)	< 0.01	86 (1.1)		173 (9.7)	111 (25.0)	< 0.01
Neoplasia	481 (4.9)	249 (4.1)	232 (6.1)	< 0.01	129 (1.7)		185 (10.4)	167 (37.6)	< 0.01
Obesity	1,626 (16.5)	955 (15.8)	671 (17.6)	0.02	1,032 (13.5)		463 (26.0)	131 (29.5)	< 0.01
Stroke	267 (2.7)	147 (2.4)	120 (3.1)	0.04	62 (0.8)		113 (6.3)	92 (20.7)	< 0.01

COPD: chronic obstructive pulmonary disease. ^a^ p value shows the differences between males and females based on bivariate analysis. ^b^ p value shows the differences between risk levels based on bivariate analysis

In the follow-up, the overall survival at 36 months was 95.2%; 477 patients with chronic conditions died (4.8%). Table 4 shows differences between survivors and nonsurvivors. Female sex was slightly higher and had a lower mean age, less immobilization at home, a lower low-risk level and a lower high-risk level, a lower complexity index, less multimorbidity and less polymedication in survivors than in nonsurvivors. Regarding differences in chronic conditions in survivors versus nonsurvivors, the mean number was 2.4 versus 4.3, and the prevalence of hypertension was 32.9% versus 69.8%, diabetes was 10.2% versus 22.9%, neoplasia was 4.1% versus 19.5%, heart failure was 1.7% versus 17.4%, dementia was 1.5 versus 15.3 and COPD was 3.5% versus 13.2%.

**Table 4:** Sociodemographic, clinician and care characteristics of the survivors and nonsurvivor chronic patients

Variables n (%)		Survivors 9,389 (95.2)	Nonsurvivors 477 (4.8)	p
Female		5,783 (61.6)	273 (57.2)	0.05
Age *		54.6 (20.3)	78.7 (16.3)	< 0.01
Age over 65-year-old		3,095 (33.0)	388 (81.3)	< 0.01
Risk Level	Low	7,459 (79.4)	179 (37.5)	< 0.01
	Medium	1,618 (17.2)	166 (34.8)	
	High	312 (3.3)	132 (27.7)	
Immobilized		172 (1.8)	128 (26.8)	< 0.01
Institutionalized		96 (1.0)	65 (13.6)	< 0.01
Primary caregiver		135 (1.4)	94 (19.7)	< 0.01
Home support		54 (0.6)	26 (5.5)	< 0.01
Palliative care		15 (0.2)	29 (6.1)	< 0.01
Complexity index *		6.2 (6.0)	16.3 (14.8)	< 0.01
No. of chronic conditions *		2.4 (1.7)	4.3 (2.5)	< 0.01
1 chronic condition 2 chronic conditions 3 chronic conditions 4 chronic conditions 5 chronic conditions > 5 chronic conditions		3,768 (40.1) 2,124 (22.6) 1,407 (15.0) 929 (9.9) 555 (5.9) 606 (6.2)	62 (13.0) 61 (12.8) 80 (16.8) 92 (19.3) 54 (11.3) 128 (26.8)	< 0.01 < 0.01 0.287 < 0.01 < 0.01 < 0.01
Multimorbidity		5,621 (59.9)	415 (87.0)	< 0.01
Arterial hypertension		3,085 (32.9)	333 (69.8)	< 0.01
Chronic heart failure		157 (1.7)	83 (17.4)	< 0.01
Chronic renal insufficiency		92 (1.0)	50 (10.5)	< 0.01
Cirrhosis		456 (4.8)	23 (4.9)	0.9
COPD		326 (3.5)	63 (13.2)	< 0.01
Dementia		140 (1.5)	73 (15.3)	< 0.01
Depression		1,165 (12.4)	86 (18.0)	< 0.01
Diabetes Mellitus		954 (10.2)	109 (22.9)	< 0.01
Ischaemic heart disease		304 (3.2)	66 (13.8)	< 0.01
Neoplasia		388 (4.1)	93 (19.5)	< 0.01
Obesity		1,558 (16.6)	68 (14.3)	0.18
Stroke		216 (2.3)	51 (10.7)	< 0.01
Polymedicated		1,306 (13.9)	292 (61.2)	< 0.01

*$\overline X$ (SD). COPD: chronic obstructive pulmonary disease. ^a^ p value shows the differences between survivors and nonsurvivors based on the bivariate analysis

*Additional file 2* shows the sociodemographic, clinician and care characteristics of the patients with chronic conditions and by sex, and *Additional file 3* shows their differences by risk levels.

**Fig. 1:**
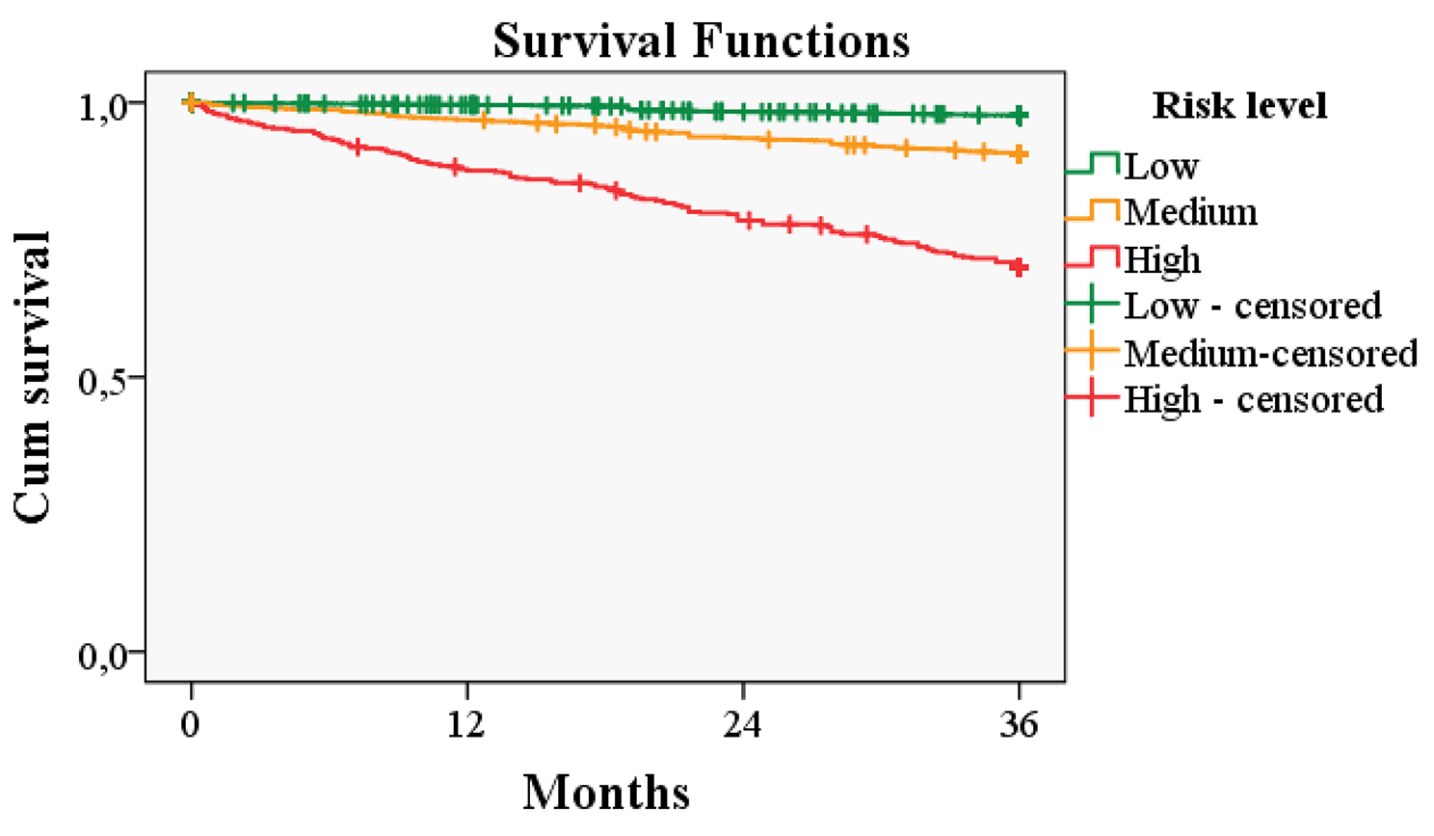
Kaplan–Meier survival curve of patients with chronic conditions according to risk level

The median survival was 36 months. Survival according to the level of risk was 97.7% in the low-risk group; 90.7% in the medium-risk group; and 70.3% in the high-risk group (Fig. 1).

The factors associated with lower survival were age ≥ 65 years (HR = 1.3; 95% CI = 1.1–1.6), receiving palliative care (HR = 3.4; 95% CI = 2.6–4.5), 5 chronic conditions or more (HR = 1.5; 95% CI = 1.2-2.0), high versus low risk level (HR = 2.4; 95% CI = 1.6–3.7), complexity index (HR = 1.03; 95% CI = 1.02–1.04) and polymedication (HR = 2.6; 95% CI = 2.0-3.3). Table 5 lists the factors associated with lower survival.

**Table 5:** Factors associated with lower survival

Variable	HR	95% CI		p	Wald
Age over 65 Palliative care ≥ 5 chronic conditions High versus medium risk level High versus low risk level Complexity index Polymedication	1.299 3.405 1.551 1.431 2.439 1.032 2.564	1.080 2.563 1.204 1.106 1.599 1.022 2.001	1.561 4.522 1.998 1.850 3.722 1.041 3.285	< 0.001 < 0.001 0.001 0.003 < 0.001 < 0.001 < 0.001	7.745 71.580 11.526 7.455 17.107 45.502 55.403

Cox multivariate proportional hazards model (n = 9,866)

HR: hazard ratio; CI: confidence interval

## Discussion

### Main findings

A total of 54.4% of the total population assigned to the primary health centre had at least one chronic pathology, 77.4% had low risk, 18.1% had medium risk, and 4.5% had high risk according to the AMG. A total of 4.8% of the patients with chronic conditions died during the three years of follow-up. Patients who survived were more likely to be female, younger, less immobilized, have less need for care, have a low risk and fewer and milder chronic conditions than nonsurvivors. The median survival was 36 months. There was a gradual and significant decrease in the survival of patients with chronic conditions according to their level of risk as defined by adjusted morbidity groups. Other factors, such as age older than 65 years, receiving palliative care, having five or more chronic conditions, higher complexity index, and polymedication, had a negative effect on survival.

### Comparison with other studies

The prevalence of patients with chronic conditions in our study coincided with the results of the 2020 European Health Survey in Spain [26], which revealed a prevalence of patients with chronic conditions in Spain of 54.3%. Studies on the distribution of patients with chronic conditions according to AMG show similar figures to those observed in the present study [12, 13, 20–22].

Regarding the characteristics of patients with chronic conditions, we observed a slight predominance of women, in line with other series [27]. Among patients with low- and medium-risk levels, there was an even greater proportion of women than among high-risk patients (61.1%, 65.0%, and 52.3%, respectively). This could be because in these strata of lower risk levels, less serious and more frequent conditions predominate among women, such as depression.

Regarding the stratification according to the AMG, most patients with chronic conditions (77.4%) had low risk, 18.1% had medium risk, and 4.5% had high risk. Although this study was conducted in a single health centre, this distribution agrees with the stratification pyramid of patients with chronic conditions in the Region of Madrid, as evidenced in the literature [13, 14].

Significant differences in survival were observed between risk levels, especially between low- and high-risk groups. These results allow us to consider that the risk levels assigned by the AMG tool are useful to predict survival at 3 years.

The great variety of concepts and definitions related to clinical complexity, as well as the existence of studies with different designs, make it difficult to compare results between studies. In a longitudinal descriptive study of 814 complex patients with chronic conditions from 40 health centres in Andalusia, the mortality at one year of follow-up was 17.8% [28], much higher than that observed in high-risk patients in our cohort at 1 year (4.3%), whose mortality did not increase significantly until the 3rd year of follow-up.

The factor that was most strongly associated with lower survival among patients with chronic conditions was receiving palliative care, followed by polymedication. Polymedication has been one of the factors most associated with mortality in other studies [29, 30]. On the one hand, polymedication is clearly related to suffering a wide range of adverse consequences, including direct and indirect health service utilization as well as increased clinical risks due to greater number of chronic conditions and more severe outcomes [30–32]. However, the association between polymedication and mortality seems to be verified independently when analyses are adjusted for confounding factors such as the number of health conditions [30, 31]. Although the treatment received is adequate, polymedication leads to a higher risk of suffering an adverse drug event and mortality for two main reasons: (a) possible errors in medication intake by older patients and (b) possible interactions between drugs [33–35]. Also, this association may be explained because polymedication reflects the need to control a more advanced and complex clinical condition or higher severity and worsening of the patient´s health conditions [36, 37]. Alternatively, a higher number of simultaneous prescriptions made by different health professionals in different care levels may reflect fragmented care that is not patient-centred, which results in inappropriate polymedication [38]. Therefore, it is necessary to emphasize the need for an integrated approach to polypharmacy and health care delivery that could balance the risks and benefits in medication prescribing [36, 37, 39].

Having  ≥ 2 chronic conditions (multimorbidity) was associated with higher mortality, although this relationship was only statistically significant for ≥ 5 chronic conditions in the multivariate analysis. These data are in agreement with a meta-analysis that concluded that there is an association between having three or more chronic conditions and higher mortality [4]. Additionally, adults with five or more chronic conditions have been associated with more social and health problems, prescriptions, health service utilization and health spending, which are also related to more complexity and higher morbidity and mortality [40–46]. However, in other studies, no association was found between mortality and the number of chronic conditions [47]. Thus, multimorbidity with a threshold of 2 or more conditions is a factor with a relatively weak impact on mortality compared to other factors, such as polymedication and high-risk level. This could be explained because the chronic conditions considered included some clinical conditions that may not always be deemed as actual chronic conditions with clinical expression but as risk factors that determine higher incidences of disease (i.e., dyslipidaemia, arterial hypertension, osteoporosis, or obesity). Consequently, using the term chronic condition, including risk factors, may not reflect a similar physical burden or the same impact on functional status and quality of life in two subjects with similar numbers of conditions. This agrees with a systematic review made by Willadsen et al. [48] in which risk factors were shown to be a reason for the high prevalence of multimorbidity and highlighted aspects concerning awareness of future illness, rather than the actual disease burden or functional status.

In the same way, the association between advanced age and mortality was evident, but with a lower impact than other factors, similar to what has been seen in other studies [28, 29]. A study of geriatric patients in Brazil (Leme et al. (2019), whose only inclusion criterion was age between 70 and 85 years, revealed a mortality at 6 years of follow-up of 21.2%, lower than that of our sample of patients with chronic conditions at 3 years [47]. However, this could be explained because age could be associated with other factors, such as functional disability and loss of quality of life, more number and severity of the conditions and polymedication, which usually increase the risk of death.

We found an association between the complexity index and mortality. Other authors have observed a decrease in survival with complexity [49], but there is no homogeneity in the measures of clinical complexity but rather a great variability of indices and scales [49]. In the AMG tool, the complexity index of the patient is taken into account as a function of morbidity (number of chronic conditions, number of organ systems affected by chronic condition(s), relevant conditions, and their complexity indices) [13] but not other factors, such as patient functionality and psychosocial problems. Different studies agree that functional capacity is one of the most important factors in the prediction of mortality risk [5, 47, 50, 51]. Loss of functional capacity and mortality increase with multimorbidity (19). Marengoni et al. (2009) found a risk of mortality 7.7 (95% CI: 4.7; 12.6) times higher among older patients simultaneously presenting multimorbidity and low functional level than individuals without these characteristics [51]. Among patients with a low functional level but without multimorbidity, mortality increased only 2.5 (95% CI: 1.6; 3.8) times over that among patients who did not present either of these two characteristics [51]. In this sense, it is possible that the use of functional status as an indicator of complexity and a predictor of mortality will replace comorbidity indices based on the number and complexity of the chronic conditions.

Similarly, there are studies that suggest that different combinations of chronic conditions are associated with marked differences in mortality [6, 29, 50, 52]. In our study, chronic condition patterns were not studied, nor were they found to affect survival.

Sex was not statistically significant in the multivariate model. This is contrary to some studies in which men had a higher mortality than women [29, 51]. Although chronic conditions associated with higher mortality were more prevalent among men, such as cardiovascular conditions, neoplasia, and COPD, the women had a higher prevalence of other factors associated with mortality, such as immobilization, multimorbidity, and polymedication. This balance between different mortality risk factors could explain this result.

### Limitations

In the design of our study, we must highlight those inherent to a secondary source of data (electronic medical records of AP-Madrid): (a) possible biases of information linked to a variability in the coding of chronic conditions and a lack of disease records; (b) unavailability of data due to limitations of the software itself, such as socioeconomic data and data on the duration and severity of chronic conditions; (c) lack of other data, as happened with the Barthel index and other capacity assessment scales, which has prevented us from analysing these aspects. Last, our study did not collect data on acute associated conditions and their interaction with chronic conditions. However, rigorous research has been carried out with these data sources, and there is validation of the diagnoses in the clinical history [20, 21]. Likewise, it should be noted that the use of secondary clinical-administrative sources for epidemiological studies makes it possible to work with almost all individuals and not with partial samples or volunteers, minimizing selection and memory biases.

### Implications

The current study supports AMG as a positive tool for the prediction of patient survival. The AMG complexity index performed properly in explaining a relevant outcome such as survival in a specific cohort of patients with chronic conditions with different risk levels, as other studies have shown before with a population-based approach [16, 17].

These findings provide policymakers, medical directors and public health researchers with evidence on the use and performance of this morbidity tool. Additionally, our data provide health professionals with new useful information regarding the relationship of AMG complexity and risk stratification with survival. This is important for clinical practice since, considering these complexity and risk levels, primary care professionals can assign a level of intervention for each individual patient in the electronic clinical record that puts in motion a set of coordinated actions between different health care areas and professionals adapted to patient care needs, as recommended in the Madrid Care Strategy for people with chronic conditions. However, more studies analysing AMG should be performed in other Spanish regions to support its use against other population stratification tools.

## Conclusions

The 3-year survival was significantly higher in chronic low- and medium-risk patients than in high-risk patients. Survival was lower among patients who had already received palliative care, polymedication, or high-risk care. Older age, five or more chronic conditions and complexity index were other factors that had a negative effect on survival. The AMG is useful in explaining survival outcomes and may be valuable for clinical practice, resource planning and public health research.

## Electronic supplementary material

Below is the link to the electronic supplementary material.

Supplementary File 1, 2 and 3.

## Data Availability

The datasets generated and analysed during the current study are not publicly available due to belonging to the Madrid Primary Care Electronic Clinical Record (AP Madrid), but are available from the corresponding author on reasonable request.
