# Supplementary material for: Adjusted morbidity groups and survival: a retrospective cohort study of primary care patients with chronic conditions
Source: BMC Prim Care. 2023 Apr 20;24:103. doi: 10.1186/s12875-023-02059-9 (PMC10120109; doi:10.1186/s12875-023-02059-9)
Supplement: Supplementary file 1 — , 2 and 3. [file 12875_2023_2059_MOESM1_ESM.pdf]

TITLE PAGE

TITLE:

Adjusted morbidity groups and survival: A retrospective cohort study of primary care patients with chronic conditions.

AUTHORS:

Mariana Bandeira-de Oliveira[1], Teresa Aparicio-González[2], Isabel del Cura-González[3,4,5], Carmen Suárez-Fernández[6,7], Ricardo Rodríguez-Barrientos[3], Jaime Barrio-Cortes[3,8,9*]

1. Ciudad Jardin Health Centre, Primary Care Management, Madrid, Spain.
2. Goya Health Centre, Primary Care Management, Madrid, Spain.
3. Research Unit. Primary Care Management, Madrid, Spain.
4. Network for Research in Health Services in Chronic Diseases (REDISSEC), Madrid, Spain.
5. Department of Medical Specialties and Public Health, Rey Juan Carlos University, Madrid, Spain.
6. University Hospital of La Princesa, Madrid, Spain.
7. Department of Medicine, Autonomous University of Madrid, Madrid, Spain.
8. Foundation for Biosanitary Research and Innovation in Primary Care, Madrid, Spain.
9. Camilo José Cela University, Madrid, Spain.

*** CORRESPONDING AUTHOR:**

Jaime Barrio-Cortes.

Email: jaime.barrio@salud.madrid.org

Telephone: 660117699

Additional file 1. Types of chronic conditions considered by the Adjusted Morbidity Group (AMG) in the Community of Madrid at the time of data extraction

Alcohol abuse
Anaemia
Aorta aneurysm
Anxiety
Arthritis
Arthrosis
Asthma
Attention-Deficit/Hyperactivity Disorder (ADHD)
Bladder cancer
Breast cancer
Cardiopulmonary disease
Central nervous system cancer
Cervical cancer
Cirrhosis
Colon cancer
Dementia
Depression
Diabetes Mellitus
Dyslipidaemia
Dysrhythmias
Ear, nose and throat cancer
Endometrial cancer
Epilepsy
Gastrointestinal ulcer
Glaucoma
Heart chronic failure
Hepatoblastoma
Hodgkin/Other lymphomas

Human immunodeficiency virus (HIV)
Hyperlipidemia
Hypertension
Ischemic heart disease
Leukemia
Liver cancer
Lung cancer
Mental retardation
Multiple sclerosis
Obesity
Obstructive chronic pulmonary disease (OCPD)
Osteoarthritis
Osteoporosis
Pancreatic cancer
Parkinson
Prostate cancer
Renal cancer
Renal chronic failure
Retinoblastoma
Schizophrenia
Skin cancer
Soft tissues cancer
Stomach cancer
Stroke
Substance abuse
Testicle cancer
Thyroid cancer
Thyroid disorder
Ulcerative colitis
Valvular heart disease
Vasculitis

Additional file 2. Sociodemographic, clinician and care characteristics of the surviving and deceased patients with chronic conditions, segmented by sex.

Variables n (%)		Survivors 9,389 (95.2)			Non-survivors 477 (4.8)		
		Female 5,783 (61.6)	Male 3,606 (38.4)	p-value[a]	Female 273 (57.2)	Male 204 (42.8)	p-value[b]
Risk Level	Low	4,556 (78.8)	2,903 (80.5)	<0.01	109 (39.9)	70 (34.3)	<0.01
	Medium	1,053 (18.2)	565 (15.7)		106 (38.8)	60 (29.4)	
	High	174 (3.0)	138 (3.8)		58 (21.2)	74 (36.3)	
Age *		56 (20.2)	52.3 (20.2)	<0.01	82.2 (15.5)	74.1 (17.3)	<0.01
Immobilized		138 (2.4)	34 (0.9)	<0.01	85 (31.1)	43 (21.1)	0.01
Institutionalized		74 (1.3)	22 (0.6)	<0.01	48 (17.6)	17 (8.3)	<0.01
Primary caregiver		107 (1.9)	28 (0.8)	<0.01	57 (20.9)	37 (18.1)	0.46
Home support		44 (0.8)	10 (0.3)	<0.01	14 (5.1)	12 (5.9)	0.72
Palliative care		9 (0.2)	6 (0.2)	0.90	11 (4.0)	18 (8.8)	0.03
Complexity index *		6.3 (0.1)	6.1 (0.1)	0.03	14.5 (12.6)	18.7 (17.1)	<0.01
No. of chronic diseases *		2.5 (0.0)	2.3 (0.0)	<0.01	4.3 (2.5)	4.2 (2.6)	0.71
Multimorbidity		3,588 (62)	2,033 (56.4)	<0.01	243 (89)	172 (84.3)	0.13
Arterial hypertension		1,795 (31)	1,290 (35.8)	<0.01	203 (74.4)	130 (63.7)	0.01
Chronic heart failure		96 (1.7)	61 (1.7)	0.91	55 (20.1)	28 (13.7)	0.07
Chronic renal insufficiency		48 (0.8)	44 (1.2)	0.07	22 (8.1)	28 (13.7)	0.05
Cirrhosis		228 (3.9)	228 (6.3)	<0.01	13 (4.8)	10 (4.9)	0.94
COPD		146 (2.5)	180 (5)	<0.01	22 (8.1)	41 (20.1)	<0.01
Dementia		111 (1.9)	29 (0.8)	<0.01	51 (18.7)	22 (10.8)	0.02
Depression		893 (15.4)	272 (7.5)	<0.01	64 (23.4)	22 (10.8)	<0.01
Diabetes Mellitus		491 (8.5)	463 (12.8)	<0.01	55 (20.1)	54 (26.5)	0.11
Ischaemic heart disease		115 (2.0)	189 (5.2)	<0.01	22 (8.1)	44 (21.6)	<0.01
Neoplasia		204 (3.5)	184 (5.1)	<0.01	45 (16.5)	48 (23.5)	0.06
Obesity		914 (15.8)	644 (17.9)	0.01	41 (15)	27 (13.2)	0.58
Stroke		124 (2.1)	92 (2.6)	0.20	23 (16.5)	28 (15.7)	0.07
Polymedicated		917 (15.9)	389 (10.8)	<0.01	184 (67.4)	108 (52.9)	<0.01

*$\bar{X}$ (SD). COPD: chronic obstructive pulmonary disease. [a] p-value is the differences between male and female based on the bivariate analysis.

Additional file 3. Sociodemographic, clinician and care characteristics of surviving and deceased patients with chronic conditions, segmented by risk level.

Variables n (%)	Survivors 9,389 (95.2%)				Nonsurvivors 477 (4.8%)			
	Low risk 7,459 (97.7)	Medium risk 1,618 (90.7)	High risk 312 (70.3)	p-value[a]	Low risk 179 (2.3)	Medium risk 166 (9.3)	High risk 132 (29.7)	p-value[a]
Female sex	4,556 (61.1)	1,053 (65.1)	174 (55.8)	<0.01	109 (60.9)	106 (63.9)	58 (43.9)	<0.01
Age *	50.1 (19.1)	70.9 (15)	75.9 (12.9)	<0.01	70.8 (21.2)	84.5 (9.9)	82.1 (11.9)	<0.01
Immobilized	33 (0.4)	82 (5.1)	57 (18.3.3)	<0.01	16 (8.9)	44 (26.5)	68 (51.5)	<0.01
Institutionalized	44 (0.6)	32 (2)	20 (6.4)	<0.01	23 (12.8)	20 (12)	22 (16.7)	0.5
Primary caregiver	19 (0.3)	67 (4.1)	49 (15.7)	<0.01	7 (3.9)	34 (20.5)	53 (40.2)	<0.01
Home support	13 (0.2)	28 (1.7)	13 (4.2)	<0.01	0 (0)	10 (6)	16 (12.1)	<0.01
Palliative care	7 (0.1)	4 (0.2)	4 (1.3)	<0.01	1 (0.6)	3 (1.8)	25 (18.9)	<0.01
Complexity index *	3.9 (2.2)	12.3 (2.6)	28.5 (10.3)	<0.01	5 (2.3)	13.5 (3.1)	34.9 (15.6)	<0.01
No. of chronic diseases *	1.9 (1.1)	4.3 (1.6)	6.7 (2.4)	<0.01	2.3 (1.1)	4.4 (1.4)	6.9 (2.5)	<0.01
Multimorbidity	3,753 (50.3)	1,558 (96.3)	310 (99.4)	<0.01	122 (68.2)	163 (98.2)	130 (98.5)	<0.01
Arterial hypertension	1,771 (23.7)	1,060 (65.5)	254 (81.4)	<0.01	84 (46.9)	139 (83.7)	110 (83.3)	<0.01
Cirrhosis	238 (3.2)	181 (11.2)	37 (11.9)	<0.01	3 (1.7)	7 (4.2)	13 (9.8)	<0.01
Chronic heart failure	12 (0.2)	75 (4.6)	70 (22.4)	<0.01	4 (2.2)	26 (15.7)	53 (40.2)	<0.01
Chronic renal insufficiency	9 (0.1)	28(1.7)	55 (17.6)	<0.01	0 (0)	8 (4.8)	42 (31.8)	<0.01
COPD	110 (1.5)	142 (8.8)	74 (23.7)	<0.01	5 (2.8)	23 (13.9)	35 (26.5)	<0.01
Dementia	45 (0.6)	64 (4)	31 (9.9)	<0.01	19 (10.6)	29 (17.5)	25 (18.9)	<0.01
Depression	739 (9.9)	355 (21.9)	71 (22.8)	<0.01	25 (14)	31 (18.7)	30 (22.7)	0.1
Diabetes Mellitus	420 (5.6)	396 (24.5)	138 (44.2)	<0.01	22 (12.3)	36 (21.7)	51 (38.6)	<0.01
Ischaemic heart disease	81 (1.1)	149 (9.2)	74 (23.7)	<0.01	5 (2.8)	24 (14.5)	37 (28)	<0.01
Neoplasia	122 (1.6)	157 (9.7)	109 (34.9)	<0.01	7 (3.9)	28 (16.9)	58 (43.9)	<0.01
Obesity	1,013 (13.6)	448 (2.7)	97 (31.1)	<0.01	19 (10.6)	15 (9)	34 (25.8)	<0.01
Stroke	57 (0.8)	98 (6.1)	61 (19.6)	<0.01	5 (2.8)	15 (9)	31 (23.5)	<0.01
Polymedicated	413 (5.5)	652 (40.3)	241 (77.2)	<0.01	60 (33.5)	122 (73.5)	110 (83.3)	<0.01

*$\bar{X}$ (DE). COPD - chronic obstructive pulmonary disease. [a] p-value is the differences between risk levels based on bivariate analysis.
